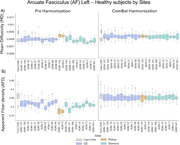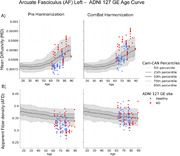# Harmonization of diffusion MRI measures is crucial for white matter tract normative assessment in ADNI

**DOI:** 10.1002/alz.083704

**Published:** 2025-01-09

**Authors:** Maxime Descoteaux, Gabriel Girard, Manon Edde, Félix Dumais, Matthieu Dumont, Jean‐Christophe Houde, Pierre‐Marc Jodoin, Jean‐Rene Belanger

**Affiliations:** ^1^ Imeka Solutions Inc, Sherbrooke, QC Canada; ^2^ Université de Sherbrooke, Sherbrooke, QC Canada

## Abstract

**Background:**

Diffusion MRI (dMRI) measures are variable across sites and MRI vendors, which leads to a *site bias* in the reported quantitative white matter metrics (Figure 1). ComBat is currently the go‐to method for harmonizing MRI data. However, to our knowledge, the harmonization power of ComBat has not been convincingly demonstrated on the ADNI cohort and was never tested in the context of normative assessment for Alzheimer’s disease (AD) patients.

**Methods:**

We select 21 sites in the ADNI cohort with at least 10 healthy controls (HC) datapoints per site. Instead of harmonizing all patients from every site onto an average template, as done in ComBat, we identified a reference site containing a large number of HC of all ages and gender. We use the Cam‐CAN dataset, with 441 subjects aged 18‐87. Moreover, instead of computing the harmonization function from every subject (HC and AD), only HC are used. The harmonization function of each site is thus computed in a pairwise fashion between each ADNI site and the Cam‐CAN site. Finally, once the harmonization function of each site is computed, all subjects of every site are harmonized towards the Cam‐CAN site. All results are shown for the Mean Diffusivity and Apparent Fiber Density dMRI measures in the left arcuate fasciculus.

**Results:**

The left panel of Figure 1 shows how dMRI measures vary across sites/vendors. The most striking bias is between Philips sites/scanners and the rest. The right panel of Figure 1 shows the successful harmonization effect of ComBat. In Figure 2, the Cam‐CAN reference is shown in gray with different percentile distributions across ages and AD patients from the GE ADNI site #127 before and after harmonization. Harmonization clearly moves HC and AD datapoints in the correct measurement “space” and preserves disease stage (HC vs AD).

**Conclusions:**

We showed that harmonization is crucial in white matter dMRI in ADNI. Harmonization methods can correct site biases and are robust to pathology, as HC and AD patients are successfully harmonized across sites. Harmonization is thus crucial for data pooling and quantitative comparison in large multi‐site datasets such as ADNI.